# 4Flu - an individual based simulation tool to study the effects of quadrivalent vaccination on seasonal influenza in Germany

**DOI:** 10.1186/1471-2334-14-365

**Published:** 2014-07-03

**Authors:** Martin Eichner, Markus Schwehm, Johannes Hain, Helmut Uphoff, Bernd Salzberger, Markus Knuf, Ruprecht Schmidt-Ott

**Affiliations:** 1 Department for Clinical Epidemiology and Applied Biometry, University of Tübingen, Silcherstr. 5, 72076 Tübingen, Germany; 2 Epimos GmbH, Uhlandstr. 3, 72144 Dusslingen, Germany; 3 ExploSYS GmbH, Otto-Hahn-Weg 6, 70771 Leinfelden-Echterdingen, Germany; 4 GlaxoSmithKline GmbH & Co. KG, Prinzregentenplatz 9, 81675 München, Germany; 5 Hessisches Landesprüfungs- und Untersuchungsamt im Gesundheitswesen, Zentrum für Gesundheitsschutz, Wolframstr. 33, 35683 Dillenburg, Germany; 6 Klinik f. Innere Medizin, Universitätsklinikum Regensburg, 93042 Regensburg, Germany; 7 Dr. Horst Schmidt Klinik, Klinik für Kinder und Jugendliche, Ludwig-Erhard-Str. 100, 65199 Wiesbaden, Germany; 8 GlaxoSmithKline Vaccines, Wavre, Belgium

**Keywords:** Influenza, Vaccination, Simulation, Mathematical model

## Abstract

**Background:**

Influenza vaccines contain Influenza A and B antigens and are adjusted annually to match the characteristics of circulating viruses. In Germany, Influenza B viruses belonged to the B/Yamagata lineage, but since 2001, the antigenically distinct B/Victoria lineage has been co-circulating. Trivalent influenza vaccines (TIV) contain antigens of the two A subtypes A(H3N2) and A(H1N1), yet of only one B lineage, resulting in frequent vaccine mismatches. Since 2012, the WHO has been recommending vaccine strains from both B lineages, paving the way for quadrivalent influenza vaccines (QIV).

**Methods:**

Using an individual-based simulation tool, we simulate the concomitant transmission of four influenza strains, and compare the effects of TIV and QIV on the infection incidence. Individuals are connected in a dynamically evolving age-dependent contact network based on the POLYMOD matrix; their age-distribution reproduces German demographic data and predictions. The model considers maternal protection, boosting of existing immunity, loss of immunity, and cross-immunizing events between the B lineages. Calibration to the observed annual infection incidence of 10.6% among young adults yielded a basic reproduction number of 1.575. Vaccinations are performed annually in October and November, whereby coverage depends on the vaccinees’ age, their risk status and previous vaccination status. New drift variants are introduced at random time points, leading to a sudden loss of protective immunity for part of the population and occasionally to reduced vaccine efficacy. Simulations run for 50 years, the first 30 of which are used for initialization. During the final 20 years, individuals receive TIV or QIV, using a mirrored simulation approach.

**Results:**

Using QIV, the mean annual infection incidence can be reduced from 8,943,000 to 8,548,000, i.e. by 395,000 infections, preventing 11.2% of all Influenza B infections which still occur with TIV (95% CI: 10.7-11.8%). Using a lower B lineage cross protection than the baseline 60%, the number of Influenza B infections increases and the number additionally prevented by QIV can be 5.5 times as high.

**Conclusions:**

Vaccination with TIV substantially reduces the Influenza incidence compared to no vaccination. Depending on the assumed degree of B lineage cross protection, QIV further reduces Influenza B incidence by 11-33%.

## Background

Before 1985, circulating Influenza B viruses were closely related to the precursor of the subsequent B/Yamagata (B/Yam) lineage [1]. Since 2001, viruses of the distinct B/Victoria (B/Vic) lineage have been detected in clinical samples from various parts of the world. The two Influenza B lineages are antigenically distinct [1-3]; genome sequence analysis revealed that they substantially differ as to the gene encoding the surface hemagglutinin [1,2] and that they continue to diverge [4,5]. Viruses of both B lineages now co-circulate together with the Influenza A viruses A(H3N2) and A(H1N1) [6]. The incidence of Influenza B infection varies substantially from year to year [6]. Surveillance data of the seasons from 2001/02 to 2010/11 show that Influenza B is responsible for 1 to 60% (mean 23%) of influenza cases in Europe and for <1 to 44% (mean 24%) in the United States, respectively [6]. Influenza B infections cause severe disease in all age groups [7] and their clinical symptoms and outcomes are similar to those of Influenza A infections [8].

Influenza vaccine design is intended to anticipate which variants will circulate in the subsequent transmission season. The antigenic composition of the vaccine is revised twice a year by the World Health Organization (WHO) and adjusted to the antigenic characteristics of circulating influenza viruses in the Northern and Southern hemisphere. Current trivalent influenza vaccines (TIV) contain antigens of A(H3N2) and A(H1N1), but of only one Influenza B lineage [9]. The efficacy of these influenza vaccines varies with the age of the vaccinees and with the degree at which the vaccines match the circulating influenza strains. In case of a good match, their efficacy can range from 70% to 90% in young adults, but it is lower in very young children and in elderly [9]. A recent meta-analysis of randomized controlled vaccine efficacy trials showed that TIV confers a 52% protection (95% confidence interval (CI): 19 to 72%) against the B-lineage not contained in the vaccine, compared to 77% (95% CI: 18 to 94%) for the lineage which matches the vaccine [10]. Co-circulation, fluctuating prevalence, and differing geographical distributions of the two B lineages have challenged global recommendations for vaccine composition [3]. In the US, the Influenza B lineage in TIV failed to match the predominant circulating B lineage in five out of ten seasons between 2001 and 2011 [11]. In 2012, WHO recommended for the first time vaccine strains from both B lineages, paving the way for the development of quadrivalent influenza vaccines (QIV) which contain strains of two B lineages and two A subtypes [12].

A variety of deterministic and stochastic models have been proposed to address various aspects of seasonal [13-16] or pandemic influenza [17-21] or to predict the size and timing of seasonal waves [22-24]. We have decided to use an individual-based stochastic model which allows for maximum flexibility in modeling immunity and vaccination strategies [25] and, thus, circumvents common oversimplifications of deterministic models [26]. We have developed the simulation tool 4Flu to compare the effect of trivalent and quadrivalent vaccination on the incidence of influenza infection in Germany. 4Flu is based on an individual-based stochastic model, which allows for the independent and simultaneous transmission of the four influenza variants A(H1N1), A(H3N2), B/Vic and B/Yam. We use the official German age-distribution for simulation years which lie in the past, and official demographic predictions for the future [27]. Individuals are connected by a dynamically changing contact network which is synchronized with the German POLYMOD contact matrix [28]. The simulation model further allows for the introduction of new drift variants and for mismatched vaccine design against new drift variants. Special care has been taken to provide a realistic immunity model which allows for maternal protection of newborns, for booster effects and waning of existing immunity and for cross-immunizing effects of infections and vaccinations. Vaccination either uses TIV or QIV. Each simulation runs for several decades and reports the annual incidence of influenza infections. Simulations are typically repeated one thousand times to average out random effects.

## Methods

We present an individual-based stochastic simulation model for the simultaneous and independent transmission of four influenza strains (A(H1N1), A(H3N2), B/Vic, B/Yam). Each simulation runs for 50 years, with each simulation year starting on July 1st: the first 10 years are used to initialize the contact network, the next 20 years for initializing age-dependent infection and immunity patterns in the population, and the final 20 years for comparing vaccines.

### Demography

As demographic changes of the population have been shown to have a major impact on the result of transmission models for seasonal influenza [13], we have made sure that the simulated demography is at all times identical to the population structure of Germany. Our simulated population corresponds to one thousandth of the German population (about 80,000 individuals). Every individual has an age and a risk status. For the years from 1993 to 2011, we use the observed age distribution of Germany, for later years, we use the official prediction “variant 1, W2” of Deutsches Statistisches Bundesamt [27]. Births and deaths occur throughout the year and are implemented such that the simulated population exactly matches the official demographic distribution at the end of each simulation year. If the size of a cohort increases from one year to the next, new individuals immigrate (see Appendix 1 for details). Depending on the age of the individuals, they are given a risk status which is used to determine the vaccination coverage and which can also be used to determine the risk of complications upon infection. As individuals age and the demography keeps changing, the individuals’ risk status is updated during the simulation to keep the specified risk fractions in the different age classes constant.

### Contact network

For the transmission of the influenza infections, we have constructed a dynamic network which connects the individuals, using the German POLYMOD matrix as summary statistic for these connections. This contact matrix is based on an EU sponsored study in which individuals were asked to supply a contact diary for one day. Contacts were then summarized in 5-year age classes [28]. To use this matrix for a population with changing demography, we connect each individual with randomly picked other individuals such that the expected number of these “outgoing” connections is proportionate to the values of the POLYMOD matrix (which depend on the ages of the individuals and their contacts). On average, each individual has a sum of 10 “outgoing” and “incoming” contacts. When individuals age, their contacts gradually change such that the population statistics of the age-dependent contact distribution remain in agreement with the POLYMOD matrix at any time (see Appendix 2 for details). In order to initialize the contact structure, we start with the German demography of 1993 and chose random contacts of individuals within 5-year age classes. We then run the simulation for 10 years without infection while keeping the age distribution constant, but allowing for births, deaths and immigrations as needed.

### Initialization of immunity, infection and vaccination

After the initialization of the contact network, a random fraction of 30% of the population is set to be immune against the Influenza A subtypes and a random fraction of 40% is set to be immune against the Influenza B lineages. To transform this crude initial immunity into a realistic age-dependent immunity pattern, transmission of influenza is simulated for 20 years before vaccination strategies are compared. As B/Vic did not circulate in Germany for at least 10 years before it re-appeared in the 2001/02 season [29], no B/Vic infections are used in the simulations before the 2001/02 simulation year. During the initialization phase from 1993 to 2012, individuals are vaccinated with TIV, using the B lineage in the vaccine which was actually used in Germany in the given year. In the 20-year evaluation period which starts with the simulation year 2013/14, individuals are either vaccinated with TIV or with QIV. As exactly the same individuals are given either one of the two vaccines on the same vaccination days, pair-wise comparisons can be made between the TIV and the QIV scenario. A 50/50 random decision is made for each future simulation year to determine which B lineage will be used in TIV.

### Natural history and transmission

For the transmission of the infection, it is not important whether a contact is labeled as “incoming” and “outgoing”; any connection of two individuals allows transmitting the infection either way. In order to calculate the probability of infecting a contact, the largest eigenvalue of the next generation matrix is calculated after initialization of the contact network. The transmission probability per contact is then given by dividing the basic reproduction number *R*_*0*_ (chosen to be 1.575 for the baseline parameter setting) by the largest eigenvalue [30] (see Appendix 3 for details). The transmission probability is further assumed to be subject to seasonal fluctuation; its maximum around Christmas is 43% higher than the baseline, its minimum occurs on 21 June of each year [31]. Throughout the year, individuals are constantly exposed to a very low rate of infection “from the outside” of the population, irrespective of their age. Infected individuals first pass through a latent period of 2 days and then become infectious for 4 days (individuals below 18 years of age) or 2 days (older individuals), respectively [32]. Transmission of the infection occurs at a random time point during the infectious period.

### Naturally acquired immunity

**Figure 1:**
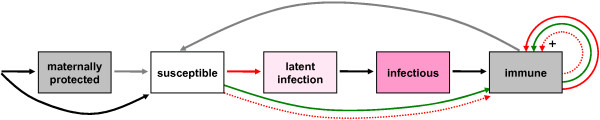
Transmission and immunity dynamics in the simulations: black arrows indicate births and disease progression, red solid arrows indicate infections, green arrows indicate successful vaccinations, and grey arrows show loss of immunity; dotted red arrows indicate cross-immunization against a B lineage caused by an infection or vaccination with the other B lineage; vaccinations and infections can also booster existing immunity (indicated by a “+”); arrows for deaths (which drain each single compartment) were omitted.

After infection, individuals acquire a temporary immunity which prevents further infections; this immunity is lost continuously at a constant rate. After losing immunity, individuals become fully susceptible again. If an immune individual gets in contact with an infectious one, or if an immune individual is vaccinated, the duration of his or her immunity can be extended by a booster vaccination or infection (see details in Appendix 4) without rendering the individual infectious. As simulation models have shown that cross-immunizing events caused by co-circulating B lineages can play an important role in the transmission dynamics of seasonal influenza [14,26], we have also incorporated this feature in 4Flu: infection with any one of the two B lineages has a 60% probability to lead to a cross-immunizing event against the other B lineage [10]. We do not consider specific cross-protection between the two Influenza A strains or among Influenza A and B, but examine the possible effect of a short-term unspecific protection in a sensitivity analysis. Newborn individuals are protected by maternal antibodies against the influenza strains against which their mother is immune (irrespective of whether her immunity was acquired by infection or vaccination). This maternal protection lasts for 2 to 4 months [33,34] and is assumed to prevent infection. The dynamics of acquiring and losing immunity is schematically depicted in Figure 1.

### Drift variants

After an average circulation time of 3.5 years (A(H3N2)) [35,36] or 7 years (all other influenza viruses), a circulating variant is replaced by a new drift variant in the simulation. For the sake of simplicity, we assume that all variants of a given influenza subtype or lineage which are introduced into the population in a simulation year belong to the same drift variant, i.e. switching to a new drift variant always occurs at the beginning of the simulation year (on July 1st). We further assume that a variant will not be introduced again after it has been replaced by a new drift variant. A percentage of 60% randomly chosen individuals who are immune against the old variant are also immune against the new one, whereas the remaining 40% are assumed to be susceptible against the new drift variant (see Appendix 6 for details) [36].

### Vaccination

**Figure 2:**
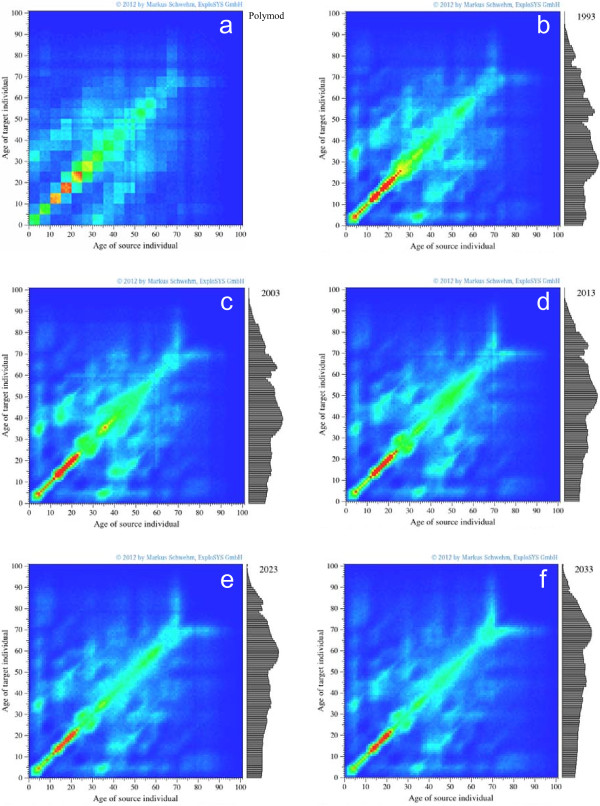
**Visualization of the contact structure in the simulated population; the number of contacts decreases from red over yellow, green, light blue to dark blue. (a)** POLYMOD contact structure before initialization of the contact network. **(b)** Contact network after a 10-year initialisation period during which the contact network is allowed to evolve while keeping the demographic structure of Germany from 1993 constant. **(c)** to **(f)** contact structure in later years. The grey bar charts on the right hand sides of the contact visualizations show the demographic distribution of Germany of the given years.

Vaccinations are performed in October and November of each simulation year [37], whereby a percentage of individuals is vaccinated, using either TIV or QIV. As shown in Table 1, the vaccinated percentage depends on the age of the individuals, on their risk status and on their vaccination status [38]: individuals who were vaccinated in the previous simulation year have twice the chance of being vaccinated again, compared to previously unvaccinated individuals (see Appendix 5 for details). An age-dependent percentage of previously susceptible vaccinees become immune after vaccination (vaccine efficacy) [39-41]. In case of TIV vaccination, we also consider the possibility of cross-immunizing effects against the B lineage which is not included in the vaccine: the age-dependent vaccine efficacy is multiplied with 0.6 to determine what percentage of vaccinees is protected against the B lineage which is missing in the vaccine. Successful vaccination can also extend the duration of pre-existing immunity (“booster-vaccination”; see Appendix 5 for details). When a new drift variant has just been introduced, the vaccine may not match this variant (vaccine mismatch). In this case, the age-dependent vaccine efficacy is multiplied by 0.6 (calculated from [39]). The decision of whether the vaccine is matched or mismatched to a new drift variant [42] is made identically for the TIV and the QIV scenario. Vaccine efficacy depends on the age of the vaccinee, but is assumed to be independent on the virus subtype or lineage. We use the same vaccine efficacy for TIV and for QIV (Table 1) [39-41], but distinguish between age classes and individual risk status [43]. For convenience, we use the same age-dependent values of the vaccination coverage in every simulation year. In the first simulation year, a total of 26.8% of the population is vaccinated. Due to demographic changes (Figure 2) and due to a high vaccination coverage in the elderly (Table 1), the overall vaccination coverage reaches 29.4% at the beginning of the evaluation period and continues increasing until the end of the simulation when it reaches 33.4%. Vaccination-derived immunity is lost much more quickly than naturally acquired immunity [44].

**Table 1:** List of parameters and baseline values

**Parameter**	**Baseline**	**Reference**
Basic reproduction number *R*_*0*_	1.575	Calibration
Maximum seasonal transmission factor	1.43	[31]
Day of maximum seasonal transmission	Dec. 21st	[31]
Duration of the latent period	2 days	[32]
External infection probability	0.0003/year	Assumed
Duration of the infectious period		[32]
- children (age 0–17 years)	4 days	
- adults (age 18 years and above)	2 days	
Duration of maternal protection	2 - 4 months	[33,34]
Immunity loss rate after infection (irrespective of influenza subtype or lineage)	1/(9.13 years)	Calculated from [31]
Average circulation time per drift variant		Calculated from [35] and [3]
- A(H1N1), B/Vic, B/Yam	7.0 years	
- A(H3N2)	3.5 years	
Vaccination coverage		[38]
- no risk group, 0.5-2 years of age	19.2%	
- no risk group, 3–6 years of age	22.4%	
- no risk group, 7–10 years of age	23.6%	
- no risk group, 11–15 years of age	11.0%	
- no risk group, 16–59 years of age	16.9%	
- no risk group, 60 years of age or older	48.8%	
- risk group, 0.5-59 years of age	33.0%	
- risk group, 60 years of age or older	64.9%	
Revaccination preference factor	2	Assumed
Probability of mismatched vaccine design when a new drift variant occurs	40%	Calculated from [3,35,42]
Vaccine efficacy (well-matched vaccine; irrespective of influenza subtype or lineage)		
- 0–1 year of age	45%	[40]
- 2–5 years of age	39%	[40]
- 6–15 years of age	69%	[40]
- 16–64 years of age	73%	[39]
- 65 years of age or older	58%	[41]
Cross protection after infection		Calculated from [39]
- percentage of individuals who are immunized against a B lineage when they are infected (or booster-infected) with the other B lineage (lineage cross protection)	60%	
- percentage of individuals who were immune against the previous drift variant, who are still protected against the new one (drift cross protection)	60%	
Cross protection after vaccination		Calculated from [39]
- vaccine efficacy multiplication factor for B lineage not contained in TIV (lineage cross protection)	0.6	
- vaccine efficacy multiplication factor for vaccinations with drift mismatch (drift cross protection)	0.6	
Immunity loss rate after vaccination	1/(1.8 years)	Calculated from [44]
Percentage of the population with elevated risk		[43]
- newborn individuals	3.0%	
- age 0 to 15 years	6.0%	
- age 16 to 59 years	14.2%	
- age 60 years and above	47.1%	

### Comparison of TIV and QIV

During the initialization period, only TIV is used as vaccine. During the evaluation period, each single simulation is run as a “TIV” and as a “QIV scenario”: (1) In order to keep the two scenarios closely matched, they use exactly the same contact network and demographic composition of the population. (2) Exactly the same individuals are vaccinated in each scenario, using either TIV or QIV at exactly the same time points. (3) If a drift event or a vaccine mismatch occurs, it occurs in the same simulation year in both scenarios. As a consequence, individuals can only differ in their epidemiologic and immunologic state with respect to the B lineages in the two scenarios. We calculate the cumulative numbers of infections for the 20 year evaluation period, using TIV or QIV and finally report the annual number of infections prevented by QIV (difference “TIV-QIV” divided by 20). As simulations are strongly influenced by random numbers, at least 1,000 simulations are run for each parameter setting and arithmetic means of the prevented number of infections are calculated.

### Model calibration

After an initial screening process, 1,000 to 2,000 simulation runs are performed for each proposed value of the basic reproduction number, starting with values from 1.5 up to 1.6, using a step size of 0.025. For each simulation, it is recorded, what percentage of individuals in the age class from 16 to 60 years experiences an influenza infection in the 2006/07 transmission period. These results are compared to the observation of Williams *et al.*[45]. As 2006/07 was labeled as a “normal” influenza season by the Arbeitsgemeinschaft Influenza [42], we used the median of the simulated infections for model calibration.

### Sensitivity analyses

The basic reproduction number *R*_*0*_ has been shown to be one of the most influential parameters which determine the outcome of influenza models [13]. Different ranges for *R*_*0*_ have been proposed in the literature (0.9-2.1 [46], 1.2-1.3 [47], 1.2-1.4 [48], 1.2-2.3 [49], 1.3-1.7 [50], 1.3-1.9 [51], 1.4-1.6 [52], 1.6-3.0 [53]). Differences in these ranges may derive from the differing epidemiologic circumstances of the places and seasons in which the estimates were derived, yet they may also be due to different ways of estimating *R*_*0*_[30,54,55]. As it has been stressed to address uncertainties in the value of *R*_*0*_[56], we perform a sensitivity analysis in which we screen the parameter range from 1.2 to 2.0, using 1,000 simulations for each value. Further sensitivity analyses address the influence of the duration of naturally acquired immunity and of the degree of B lineage cross protection.

## Results

Figure 2 shows how the contact matrix changes over time: Figure 2a shows the state of the contact network before initialization: the 5-year age classes of the German POLYMOD matrix are slightly disturbed by the German demography, but are still clearly visible. After about five years of initialization, the contact structure has changed to mostly allocate contacts to individuals of the same age, although (by construction of the algorithm) the summary statistics of the number of “outgoing” contacts per 5-year age group remain identical to the POLYMOD matrix. After ten years of network initialization, the contact network has converged to a steady state (Figure 2b). After that time, the immunologic and epidemiologic initializations are started and the demography of the simulated population is allowed to change according to the official German demography. The resulting age distributions of the German population are shown on the right hand sides of the contact matrix visualizations in Figure 2b-f.

**Figure 3:**
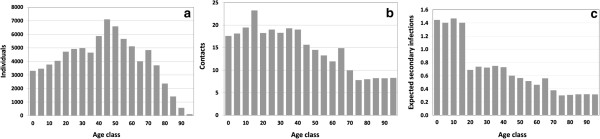
**Demographic and network properties in a randomly picked simulation on 23 September 2012 (i.e. at the end of the initialization period), a day of the year on which the seasonal variation factor is 1.0 (i.e. the transmission potential reaches its all year average).** This day precedes the annual vaccination period and the main transmission period. **(a)** Demographic structure of Germany; **(b)** average number of contacts per individual; **(c)** expected number of individuals who are infected by a single case whose age is given on the horizontal axis (averaged over the 4 influenza strains; the high number of expected infections caused by children derives from their long infectious period, their high connectivity and the immunity status of their contacts).

As the number of children keeps declining and the number of elderly grows in the German population, the frequency of contacts becomes less focused on children and young adults and tends to be more spread out over the ages. In 2012, contacts are most frequent in juveniles and then decline over age (Figure 3b). Taking into consideration the longer duration of infectiousness of children and juveniles and considering the susceptibility status of their contacts, we can calculate how many individuals are infected on average by a single infectious individual of a given age as shown in Figure 3c. The median time until an infected individual passes on the infection (generation period) is 3.3 days in our simulations.

**Figure 4:**
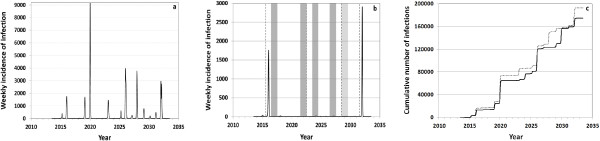
**Results of a randomly selected simulation. (a)** Weekly influenza infection incidence (all strains and lineages) in the TIV scenario. **(b)** Weekly incidence of B/Yam infections only; years in which B/Yam is not contained in TIV have a dark grey background; the introduction of new drift variants is shown by dashed vertical lines; in 2028, the vaccine is mismatched to the newly introduced drift variant (light grey background). **(c)** Cumulative number of all influenza infections which occur in the TIV scenario (dashed) and in the QIV scenario (solid line). All three graphs relate to the same simulation.

Our model is calibrated to observed data from Germany. As our simulation results vary strongly (coefficient of variation CV = 91.5% for *R*_*0*_ = 1.575), we use the median of the simulated incidence when evaluating simulation results: for each value of the basic reproduction number *R*_*0*_ between 1.5 and 1.6 (step size 0.025), we ran 1,000 to 2,000 simulations and calculated the median of the infection incidence in the 2006/07 transmission season for young adults. A basic reproduction number of *R*_*0*_ = 1.575 yields a median infection incidence of nearly exactly 10.6% as was observed among German health care workers and controls in that season [45]. Figure 4a shows the large fluctuation of annual influenza waves in a single simulation in which TIV is used throughout, whereby all infections with the two A strains and the two B lineages are added up. Figure 4b selectively shows the incidence with B/Yam for the same simulation. Years in which B/Yam is not contained in TIV are shown as dark grey bars. New B/Yam drift variants are introduced in 2015, 2022, 2028 and 2031, shown as vertical dashed lines. In 2028, the B/Yam lineage in the vaccine does not match the newly introduced drift variant, indicated by a light grey background. During two seasons, large B/Yam outbreaks occur, dwarfing the smaller waves in the other seasons. Starting with the year 2013, simulations branch into a TIV scenario and a QIV scenario (Figure 4c). The cumulative numbers of infections are calculated in each of these scenarios and are used to calculate the average annual number of infections prevented by QIV as compared to TIV.

**Figure 5:**
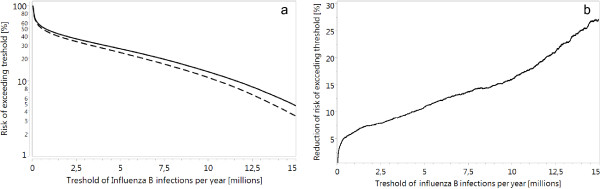
**Probability of extreme influenza years. (a)** Risk that the number of Influenza B infections per year in Germany exceeds the threshold given on the horizontal axis (solid line: TIV vaccination; dashed line: QIV vaccination). **(b)** Relative reduction in the risk of exceeding the threshold if QIV is used instead of TIV. Evaluation of 400,000 simulation years (10,000 simulations of 20 years for TIV and for QIV).

Averaged annual simulation results for the baseline set of parameter values are given in Table 2. As the simulated population is exactly one thousandth of the German population, these results have to be multiplied with 1,000 to obtain estimates for the whole of Germany. The annual number of influenza infections in Germany can be reduced from 8,943,000 to 8,548,000, i.e. by 395,000 infections per year without increasing the vaccination coverage if QIV is used instead of TIV. The prevented infections constitute 4.3% (95% confidence interval (CI): 4.1-4.5%) of all influenza infections which still occur under TIV vaccination. The simulated fraction of Influenza B infections among all infections varies widely from one season to the next; even if averaged over the 20 years of each simulation, the 95% reference interval for the percentage of Influenza B infections among all influenza infections ranges from 24.6 to 51.3% (median 37.3%) in the TIV scenario, and from 21.3 to 49.1% (median 34.3%) in the QIV scenario. Switching from TIV to QIV prevents on average 11.2% (CI: 10.7-11.8%) of all Influenza B infections which still occur under TIV vaccination. In 53% of 10,000 simulation years, the B lineage not contained in TIV caused more infections than the B lineage which was contained in TIV.Additionally to lowering the expected number of infections, QIV furthermore reduces the number of seasons with rather extreme Influenza B outbreaks (Figure 5).

**Table 2:** Average annual results (with 95% confidence intervals) obtained for the 20 year evaluation period (calculated from 2,000 simulations using the baseline parameter values) in a simulated population of about 80,000 individuals

**Age group**	**Annual mean number of infections using TIV**	**Annual mean number of infections using QIV**	**Annual mean number of prevented infections**	**Percentage prevented**
0-15	2152	2073	79	3.6
	(2140–2164)	(2061–2085)	(75–83)	(3.4-3.8)
16-60	5470	5247	223	4.0
	(5433–5506)	(5211–5282)	(212–235)	(3.8-4.2)
61+	1321	1228	93	6.9
	(1311–1331)	(1219–1237)	(90–97)	(6.7-7.2)
all	8943	8548	395	4.3
	(8885–9001)	(8491–8604)	(376–414)	(4.1-4.5)

The risk that in Germany more than 10 million Influenza B infections occur in a single transmission season is about 13.4% for the TIV scenario and 11.2% for the QIV scenario (Figure 5a), meaning that the expected duration between such extreme events can be increased from 7.5 years (TIV) to 8.9 years (QIV). For 15 million infections per year, the difference is even more pronounced (21.3 years for TIV, 29.2 years for QIV). The risk of strong Influenza B seasons can be reduced by up to 27% if QIV is used (Figure 5b).

**Figure 6:**
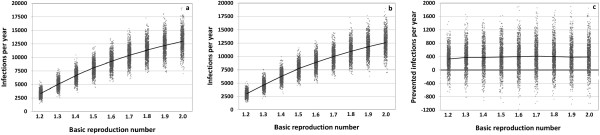
**Results of a sensitivity analysis in which the basic reproduction number is varied from 1.2 to 2.0.** For each value, 1,000 simulations with about 80,000 individuals are run for 20 years. Average annual number of infections for **(a)** the TIV scenario and **(b)** the QIV scenario. **(c)** Average number of infections prevented per year by QIV. Mean values are connected.

We address parameter uncertainty in various sensitivity analyses: the basic reproduction number, *R*_*0*_, is varied from 1.2 to 2.0 (baseline value: 1.575) while the values of the other parameters are kept at their baseline values. As *R*_*0*_ summarizes the contagiousness of the disease, the average annual number of infections in Germany grows with increasing *R*_*0*_ from 3,354,000 to 12,973,000 per year in the TIV scenario, and from 3,029,000 to 12,587,000 in the QIV scenario (Figure 6a-c and Appendix 8); the average number of infections annually prevented by QIV rises from 325,000 infections for *R*_*0*_ = 1.2, to about 386,000 infections for *R*_*0*_ = 1.5, and, thereafter, remains about constant on a level of 395,000 infections. Due to the rising denominator, the percentage of prevented infections falls with increasing *R*_*0*_ from 9.5 to 3.0% (all infections), or from 21.6 to 7.8% (Influenza B infections).

**Figure 7:**
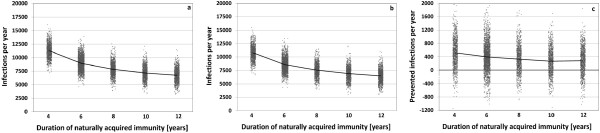
**Results of a sensitivity analysis in which the immunity loss rate is varied such that the expected duration of naturally acquired Influenza A(H1N1) immunity lasts 4 to 12 years.** For each value, 1,000 simulations with about 80,000 individuals are run for 20 years; 2,000 simulations for the baseline duration of 6 years. The same rate is applied to all influenza infections leading to the same average duration for B/Vic and B/Yam, but to a shorter duration for A(H3N2), due to the more frequent appearance of drift variants (see Appendix 4 for details). Average annual number of infections for **(a)** the TIV scenario and **(b)** the QIV scenario. **(c)** Average number of infections prevented per year by QIV. Mean values are connected.

In a further sensitivity analysis, the rate at which immunity is lost is varied such that the duration of naturally acquired immunity lasts on average 4 to 12 years against A(H1N1) and the B lineages, and for 3.2 to 7.1 years against A(H3N2) (see Appendix 4 for details). The average annual number of infections drops with increasing immunity duration from 11,267,000 to 6,735,000 infections per year in the TIV scenario, and from 10,751,000 to 6,453,000 in the QIV scenario (Figure 7a-c and Appendix 8). The average number of infections annually prevented by QIV correspondingly drops from 517,000 (4.6% of all infections, or 11.7% of influenza B infections) to 282,000 infections (4.1% of all infections, or 10.7% of influenza B infections).

Following the parameter choice of Vynnycky *et al.*[31], we perform a sensitivity analysis in which we only increase the duration of naturally acquired Influenza B immunity from the baseline value of 6 years to 12 years while keeping the immunity duration of Influenza A at the baseline value. This reduces the annual number of infections from baseline 8,943,000 to 8,098,000 in the TIV scenario, and from baseline 8,548,000 to 7,814,000 in the QIV scenario. The average number of infections annually prevented by QIV correspondingly drops from baseline 395,000 (4.3% of all infections, or 11.2% of all Influenza B infections) to 284,000 infections (3.5% of all infections, or 10.8% of all Influenza B infections). Increasing the duration of Influenza B immunity shifts the 95% reference intervals of the percentage of Influenza B infections among all influenza infections towards lower values, reaching 16.3-46.6% (median 30.5%) for the TIV scenario, and 13.7-43.3% (median 27.5%) for the QIV scenario.

**Figure 8:**
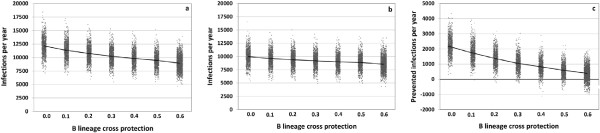
**Results of a sensitivity analysis in which the lineage cross protection is varied.** For each value, 1,000 simulations with about 80,000 individuals are run for 20 years; 2,000 simulations for the baseline value of 0.6. Average annual number of infections for **(a)** the TIV scenario and **(b)** the QIV scenario. **(c)** Average number of infections prevented per year by QIV. Mean values are connected.

Another highly influential parameter is the degree of B lineage cross protection: in a sensitivity analysis, we increase the percentage of individuals who are immunized against an Influenza B lineage when they are successfully vaccinated or infected with the other B lineage, using values from 0% up to the baseline value of 60% (Appendix 8). The higher the probability of B lineage cross protection is, the fewer Influenza B infections occur (Figure 8a-c). Whereas for the baseline value, the median percentage of Influenza B infections among all infections is 37.3% (TIV scenario), it increases to 54.1% if no B lineage cross protection is considered. Accordingly, the number of infections which can annually be prevented by QIV increases from 395,000 (baseline value) up to 2,180,000 infections if lower values are considered. If the degree of B lineage cross protection decreases over time from initially 60% to a lower value which is reached at the end of the simulation, the additional benefit of using QIV increases from the baseline result of 395,000 infections per year (obtained for a constant level of 60% B lineage cross protection) to 559,000 (decreasing the cross protection from 60% to 50%), to 706,000 (40%) and to 853,000 (30%), respectively (average values of 1,000 simulations for each parameter set). Our model does not explicitly consider cross immunity between the A strains or among Influenza A and B, but we have run a sensitivity analysis where we consider a short-term unspecific immunity which prevents infection with any influenza virus within the first weeks following an infection. As shown in Appendix 8, such an indirect protection, which may last for one to four weeks, has only a marginal effect on the results. If anything, it may slightly increase the difference between the QIV and the TIV scenario.

In order to keep the age distribution of “outgoing contacts” synchronized to the POLYMOD contact matrix despite demographic changes, contacts have to be removed or added daily. Although we keep the number of changes to a minimum, only 0.36% of the childhood contacts of 10 year old children at the beginning of the simulation are still among their contacts at the end of the simulation when they are 50 years old. We have examined the influence of contact turnover on our results in a sensitivity analysis in which we have either increased the contact turnover by replacing at random 10% of all contacts each year, or decreased contact turnover by updating contacts only once a year instead of daily (resulting in larger and longer lasting deviations of the simulated age-dependent contact distribution from the POLYMOD matrix). The effect of contact turnover on the number of infections additionally prevented by QIV is marginal (Appendix 8).

## Discussion

Our results indicate that annually an average number of 395,000 influenza infections (representing 11.2% of all Influenza B infections) can additionally be prevented without increasing vaccination coverage by switching from TIV to QIV. Although the composition of TIV during the evaluation period of our simulations is determined at random, our finding that in 53% of seasons, the B lineage not contained in TIV causes more infections than the B lineage which actually is contained in the vaccine is practically identical to findings from the US (the Influenza B lineage in TIV failed to match the predominant circulating B lineage in five out of ten seasons between 2001 and 2011) [11]. It had to be expected that this percentage should even be higher if the vaccination coverage was higher, yet this finding frequently is regarded as an indicator of having chosen the wrong B lineage in TIV.

As QIV is designed to additionally prevent Influenza B infections, our results strongly depend on the assumptions made on the transmissibility and on the duration of acquired immunity against Influenza B. Cross immunization after infection with an Influenza B lineage or after successful vaccination with TIV increases Influenza B immunity in the population. As we show in sensitivity analyses (Figure 8a-c and Appendix 8), our baseline value of 60% lineage cross protection leads to a highly conservative estimate of the effects of QIV. Using lower cross protection values, the additional benefit of using QIV instead of TIV can be up to 5.5 times as high as the baseline result of preventing 395,000 infections per year (Figure 8a-c). As the antigenic overlap of the two B lineages is clearly disadvantageous to their transmission, mutations which increase the genetic difference between the two lineages and, thus, reduce the degree of cross protection should be selected for. To check the effect of this hypothetical antigenic drift, we have continually lowered the degree of B lineage cross protection over time in a sensitivity analysis. If the degree of B lineage cross protection drops from initially 60% to finally 30%, the additional benefit of using QIV increases up to an average of 853,000 infections per year. It must be noted that we restrict cross-immunizing vaccine effects to TIV and omit them when individuals receive QIV, because it has been shown that increasing antigen amount does not enhance the efficacy of influenza vaccines [57,58] and because there is no convincing evidence that increasing the amount of antigen enhances the efficacy of influenza vaccine [59].

The simulated absolute and relative numbers of Influenza B infections fluctuate strongly from year to year. Even if they are averaged over 20 simulation years, the percentage of Influenza B infections varies from 24.6 to 51.3% (95% reference interval in the TIV scenario). This range is contained in the range of reported values on clinical Influenza B cases, yet the median percentage of Influenza B infections (37.3%) is higher than the reported average of 23% of clinical cases who are caused by Influenza B [6]. This difference between the simulated percentage of Influenza B infections and the reported percentage of Influenza B cases may partly be due to a different fraction of Influenza A and B cases being reported: it has been shown that Influenza A infections more frequently lead to high fever which may lead to more doctoral visits and finally to more case reports [60]. During a mixed A(H3N2) and B season in the Netherlands, the proportion of laboratory confirmed Influenza A was higher in hospital samples than in GP sentinels [61]. This indicates an underestimation of the incidence with types which tend to cause mild illness when laboratory data include hospital samples. With increasing severity, the percentage of strains which cause milder infections should increasingly be underestimated (e.g. from diagnosis of acute respiratory infections, ARI, to diagnosis of influenza like illness, ILI, or hospitalization). Influenza infections are diagnosed by using Flu-rapid tests. Most of these tests can identify Influenza A and B, but the proportion of selective tests which only test for Influenza A is unknown. This might cause a slight underestimation of the true Influenza B incidence. Vynnycky *et al.*[31] use in their simulation study different immunity durations for Influenza A (6 years) and Influenza B (12 years). We have addressed this in a sensitivity analysis in which we double the duration of Influenza B immunity (unlike Vynnycky *et al*. we use the resulting duration of 12 years only for naturally acquired immunity and not for immunity conferred by vaccination). As a consequence, the median percentage of Influenza B infections among all influenza infections drops to 30.5% in the TIV scenario, and the average number of infections prevented by QIV drops to 284,000 per year (preventing 3.5% of all influenza infections, or 10.8% of all Influenza B infections, respectively). If, on the other hand, we assume that the B lineage cross protection is less than 60%, the number of Influenza B infections increases, and, consequently, QIV can prevent more infections (Figure 8a-c). Without any B lineage cross protection, we expect to see more Influenza B than A infections (54.1 vs. 45.9%) in the TIV scenario, because TIV only immunizes against one B lineage, but against both A subtypes. In that extreme case, QIV would additionally prevent over two million infections per year. Although the annual incidence of infections strongly depends on *R*_*0*_, the number of prevented infections is nearly constant for *R*_*0*_ > 1.5.

The seasonal influenza waves depicted in Figure 4a show a much higher variability than in the country-wide statistics published by the Robert Koch Institute [42]: the coefficient of variation of the simulated annual number of influenza infections is in the order of 100% (TIV scenario). This high variability mainly results from using a population size of only one thousandth of the German population: geographic differences in immunity patterns and transmission reduce the variability of the reported influenza incidence. When adding up the annual incidences of ten randomly picked simulations (to mimic the combined result of ten locations with independent transmission), the coefficient of variation reduces to about 30%. In reality, much more locations ought to be combined, but their epidemiology must not be assumed to be independent.

Various contact networks have been proposed to study the spread of influenza [62-65]. In our simulations, we use a novel contact network which is based on contact information gathered for the German population [28], yet which allows for dynamic changes during the ageing process of the population. As was shown in a sensitivity analysis, the network turnover had only negligible effect on the difference between the TIV and QIV scenario. It has been suggested to use bipartite networks which explicitly consider the place of contact (school, work place, household) [66]. Although the POLYMOD data include some information on the place of the contact [28], many additional assumptions would be required to explicitly consider contact place in our simulations. Some of the contacts which regularly occur at school or work place [67] are covered by the age-dependency of contacts in the underlying POLYMOD matrix [28], but our contact network may be too rigid to allow for random encounters (e.g. during public transportation).

In our simulation studies, we make several simplifying assumptions: (1) the basic reproduction number is assumed to be equally high for all four influenza strains; differences between Influenza A and B are only due to the higher drift frequency of A(H3N2), to the missing B lineage in TIV, and to B lineage cross protection. (2) Infected children are as contagious as adults, yet they have a longer infectious period (cf. Table 1) and have more contacts than adults - specifically with other children, resulting in a larger number of secondary infections (Figure 3c). (3) We apply the same reduction factor 0.6 to the vaccine efficacy which cross-protects against the B lineage which is not contained in TIV, and to a vaccine design mismatch; likewise, we assume that only 60% of previously immune individuals are immune against a newly introduced drift variant. Tricco *et. al.*[10] report for vaccinees from 18 to 49 years of age that the vaccine efficacy against Influenza B is 77% if the lineage is contained in TIV and 52% if it is not contained in TIV, respectively. The ratio 52/77 = 0.675 is similar to our ratio of 0.6, calculated from a Cochrane review for any type of mismatch. Langley *et al*. show in a vaccination study with children that the sero-conversion rate with respect to the B lineage which is not contained in TIV is half as high as the sero-conversion rate with respect to the lineage which is contained in the vaccine [59] (cf. Appendix 6). Using a B lineage cross protection of only 50%, leads to a larger than baseline average annual number of infections prevented by QIV (Figure 8c). (4) School holidays have been shown to have a strong impact on the transmission of influenza [62], but we did not want to further complicate our model by changing contact patterns during such time periods, as different school holiday periods are used in different German states and as they change from year to year. We also do not distinguish between week days and weekends in our simulation. Using averaged social contacts throughout the week has been shown not to affect the major outcome of influenza simulation studies [63]. (5) Although vaccination coverage in Germany varies strongly between different locations and may also slightly vary over time [68], we use a constant age-dependent vaccination coverage throughout our simulations [38] which is assumed to represent a country-wide average. (6) We do not explicitly consider human travel in our simulations [20,21,47], but we apply a very small “external” infection rate which is present throughout the year. (7) Individuals who were vaccinated against A(H1N1) pdm09 in 2009 were more likely to be revaccinated in 2010 with an odds ratio (OR) of 6.02 in The Netherlands [69], yet in our simulations, the OR of revaccination ranges from 2.2 to 6.5 (for details, see Appendix 5). If the reported OR is also representative for non-pandemic years in Germany, too few individuals are revaccinated in our simulations and, thus, the effects of vaccinations may be overestimated. (8) We use the same average immunity duration after booster infections as after infections of previously susceptible individuals as it is not known whether this immunity duration should be larger or smaller than after infection. (10) Although great care has been given to use realistic assumptions on the immunity acquired by infection, the parameters associated with immunity are quite uncertain. We have addressed this uncertainty in various sensitivity analyses, which explore the effect of the duration of immunity and the degree of heterologous immunity between the B lineages. It has been difficult to obtain quantifiable evidence for cross-immunizing effects between the A strains or between Influenza A and B, yet an indirect protection may be caused by an increased production of interferons, tumor necrosis factor alpha and beta and other cytokines as has been reported after infection with influenza and various other viral infections [70-73]. As we did not want to incorporate non-influenza infections, we have restricted the study on the effects of unspecific protection to a sensitivity analysis which showed only a minor effect of indirect protection (Appendix 8).

## Conclusions

4Flu is a novel simulation tool which describes the spread of influenza viruses using a dynamically evolving contact network, reflecting German demography, with different vaccinations strategies. The results indicate that the current vaccination with TIV in Germany, although covering less than 30% of the total population [38], substantially reduces the number of influenza infections compared to no vaccination. Replacing TIV with QIV would further reduce Influenza B incidence by 395,000 to 2,180,000 influenza infections per year, depending on the degree of B lineage cross protection.

## Appendix 1 Dynamic changes in demographic structure and risk status

The observed and predicted demographic distributions of Germany are given in 1-year cohorts for every year until 2060, together with annual birth rates [27]. Our simulations start with the demographic distribution of 1993. Each individual is given a random birth day; in each simulation year, individuals are added according to the predicted number of births. If the number of individuals of a cohort declines from one year to the next, individuals are chosen to die during the simulation year; if it increases, individuals are introduced as “immigrants”. Birth, death and immigration take place at random times during the year. On each birthday, an individual’s age grows by one year. Individuals are also given a risk status which influences their probability of being vaccinated and which may influence the clinical outcome of an infection. Some individuals have an increased risk when they are born, others acquire a risk status over time. In order to avoid sudden transitions from one age group to the next one, we assume that the percentage with risk changes linearly within each age group, whereby we keep the total percentage in the age group identical to the literature value. Due to births, ageing and death, the risk fractions in the simulations keep drifting away from the specified values given in Table 1. At each birthday, it is checked whether there are currently too many or too few individuals with risk status in the cohort of the individual who is now one year older; if needed, the individual’s risk status is changed in order to reduce this deviation.

## Appendix 2 Dynamic changes in contact structure

In order to transmit the infection from one individual to another one, individuals are connected in a dynamic contact network. The average number of contacts depends on the age of the individual and on the ages of the contact persons, and is given by the POLYMOD matrix [28]. Before initializing the contact network, each individual “initiates” on average 5 contacts with others, whereby the age distribution of these contacts is proportional to the numbers given by the POLYMOD matrix. These contacts are classified as “outgoing contacts” for the source individual and as “incoming contacts” for the contacted individual (i.e. on average, every individual has 10 contacts). For each newborn individual, a random person of the age-group from 20 to 40 years is chosen (denoted as the “mother”) who initiates a contact to the newborn individual. If the mother is connected to other individuals (which usually is the case), the newborn initiates a contact to the individual whose age is closest to that of the mother; thus newborns are initially connected in triangles. Immigrants are incorporated into the existing contact network in the same way as newborns. Due to births, immigrations, ageing and deaths, the contact distribution of “outgoing” contacts keeps drifting away from the values specified by the POLYMOD matrix. In order to correct this, the distribution of “outgoing” contacts is compared at the end of each simulation day with the POLYMOD matrix assuming a population average of 5 “outgoing” contacts per individual. If too many contacts between two age groups exist, superfluous contacts are chosen at random and removed; if too few contacts exist, random individuals are chosen from the required age groups and connected. In a sensitivity analysis this “repair mechanism” is only performed annually instead of daily (resulting in lower network turnover); or an additional random percentage of contacts is removed and replaced by new random contacts (resulting in higher network turnover). In order to let the contact network adjust to the demographic structure of Germany, we run the simulation for 10 years (“contact network initialization”) without changing the demographic structure of Germany before introducing immunity and infections in the simulations.

## Appendix 3 Transmission dynamics and basic reproduction number

An infected individual does not necessarily infect all of his or her contacts; the expected number of secondary infections in a completely susceptible population is given by the basic reproduction number *R*_*0*_. In order to calculate the infection probability per contact from the basic reproduction number, the “next generation matrix” is calculated which combines the contact information in the population and the duration of contagiousness [32]. As we start the simulations with 80,976 individuals, the contact network can be described by an 80,976 × 80,976 matrix which is mostly filled with zeros. The next generation matrix is defined by the product of the network matrix and the average duration *D*_*a*_ of contagiousness (which depends on the age *a* of the infectious individual). The largest eigenvalue *E* of this matrix is calculated by power iteration (Von Mises iteration). The probability that a given contact of an infectious individual of age *a* is infected by that individual is then given by *p*_*a*_ *= R*_*0*_*D*_*a*_*/E*[30]. During the first 5 years of the initialization of the contact structure, the largest eigenvalue of the next generation changes markedly, but after that it remains stable, indicating that the contact network has converged to a steady-state. As influenza transmission is assumed to vary seasonally, we multiply the transmission probability with a seasonal factor: *p*_*a*_*(1 + 0.43cos(2π(t-173)/365))*. The seasonality is chosen such that the transmission reaches its maximum around Christmas where it is 43% higher than the average value [31]. When an individual becomes contagious, a random decision is made for each contact whether an infection event occurs. Such an event can either lead to the infection of a susceptible individual or booster existing immunity. The time point of infection is sampled at random from the infectious individual's period of infectiousness, assuming a uniform distribution. Additional to the transmission of infection between individuals, all individuals (irrespective of their age or immunity status) are constantly exposed to a very low infection rate from the “outside” of the population. Transmission of the four influenza variants occurs independently, thus, on very rare occasions, individuals may be infected with more than one variant at the same time.

The basic reproduction number of the model is calibrated such that the median percentage of young adults (16 to 60 years of age) who are infected during the simulation year 2006/07 closely matches the observed value of 10.6% of German health care workers and controls who had a serologically confirmed influenza infection or at least a fourfold rise in influenza antibody titer during the 2006/07 season [45]. The chosen baseline value of the basic reproduction number of 1.575 yields a median infection rate of 10.6% (calculated from 2,000 simulations).

## Appendix 4 Acquisition and loss of immunity

Immunity is assumed to be lost at a constant rate, leading to an exponentially distributed duration of immunity. The average duration of immunity is shorter if the immunity is caused by vaccination than when it is caused by infection. Immunity can be boostered by another infection or by a successful vaccination. If a booster event occurs, a new exponentially distributed duration of immunity is calculated for the individual. If the new duration is longer than the existing immunity, the individual’s immunity is extended accordingly. Although we use the same average length of immunity when calculating the new “booster immunity” duration, using the maximum of the existing and the new immunity duration automatically leads to a larger average duration of immunity after a booster infection than after a non-booster infection. If an individual is infected or successfully vaccinated with a virus of one of the B lineages, a cross-immunizing event can occur which protects the individual also against the other B lineage (this is determined by calculating a random number). Such cross-immunizing events can immunize susceptible individuals or booster existing immunity. In most of our analyses, we keep the percentage of B lineage cross protection constant, but in a sensitivity analysis, we allow it to decrease linearly over time, starting with a value of 60% in 2001 when B/Vic is introduced and reaching the final value of e.g. 30% on the last simulation day.

A single infection with Influenza A(H1N1) is assumed to result in an immunity which lasts on average for 6 years [31]. As we assume that new A(H1N1) drift variants appear on average every 7 years and that 60% of the old immunity is active against new drift variants (see Appendix 6 for details), immunity loss due to drift alone would, therefore, result in an average immunity duration of 7 years/(1–0.6) = 17.5 years. In order to obtain an average immunity duration of 6 years, we assume that immunity is constantly lost at a rate λ while no drift variant is introduced. The average immunity duration *D*_*I*_ is then given by *D*_*I*_ *= ((1–0.6)/7 + λ)*^*−1*^. The value *λ* = 1/(9.13 years) which yields an average duration of 6 years is used for all four influenza strains (due to the higher drift frequency, the average immunity duration against A(H3N2) results in about 4.5 years).

The average duration of immunity after vaccination is calculated from a figure given by Ruf *et al.*[44] in which the sero-protection rate (SPR) (i.e. the fraction of vaccines with a heamagglutination inhibition titer of ≥ 40) is shown for various time periods after vaccination. Values of the percentage of vaccinees who were protected *t* months after vaccination were read from a figure using Aotsuka’s freeware package “Data Picker” M: (Shareware Version 1.2. http://hp.vector.co.jp/authors/VA019223/). We obtain the parameters of the function *p*_*0*_ *+ (100-p*_*0*_)_*_VE_*_ *e*^*-αt/12*^ by least squares fit to the picked values. The SPR of vaccinees who were immune before vaccination (*p*_*0*_) is estimated to be 28.6% (95% CI from 23.5 to 33.7%), the fraction of individuals who were unprotected before vaccination, yet become protected by vaccination (VE) is estimated as 92.2% (95% CI from 84.9 to 99.6%), the immunity loss rate *α* is estimated to be 0.553 per year (95% CI from 0.394 to 0.729). The average duration of vaccination derived immunity (in the absence of drift) is given by *1/α* = 1.81 years (95% CI from 1.37 to 2.54 years).

Each newborn individual inherits maternal antibodies against all influenza strains or lineages against which his or her mother is immune at the time of birth; maternal antibodies protect the newborn individual against infection for some months. The immunological and epidemiological statuses of immigrants also need to be initialized: this is done by choosing a random individual of similar age (preferably in the year cohort of the immigrant) and transferring his or her immunity status and infection status to the immigrant. In a sensitivity analysis, we additionally consider the possibility that infection causes a short-term unspecific protection which temporarily prevents infection with any one of the influenza viruses.

## Appendix 5 Vaccination coverage

The vaccination coverage depends on the age and the risk status of individuals. Furthermore, people who were vaccinated in the previous year are more likely to be vaccinated again. The baseline revaccination factor *f*_*rev*_ of 2 means that previously vaccinated individuals are twice as likely to be chosen for vaccination; it can be interpreted as the “relative risk” (RR) of re-vaccination given a previous vaccination. At the beginning of each simulation year, each individual is assigned a “vaccination day”, irrespective of whether this individual will be vaccinated or not. The vaccination decision will be made on that day. If a percentage *c*_*a,r*_ of individuals of age *a* and risk category *r* is intended to be vaccinated in a simulation year, we first calculate the expected number of vaccinees *c*_*a,r*_*N*_*a,r*_, whereby *N*_*a,r*_ is the number of individuals in the population with age *a* and risk status *r*. If there are *N*^*+*^_*a,r*_ previously vaccinated and *N*^*−*^_*a,r*_ previously unvaccinated individuals, we sample *(c*_*a,r*_*N*_*a,r*_)_*_(f_*rev*_*N*^*+*^_*a,r*_*)/(N*^*−*^_*a,r*_ *+ f*_*rev*_*N*^*+*^_*a,r*_*)* previously vaccinated and *(c*_*a,r*_*N*_*a,r*_)_*_N^*−*^_*a,r*_*/(N*^*−*^_*a,r*_ *+ f*_*rev*_*N*^*+*^_*a,r*_*)* previously unvaccinated individuals to be vaccinated. During the vaccination period, it is continuously memorized how many individuals of each combination of age group, risk status and previous vaccination status have already been vaccinated and how many of them have already made the “decision” not to be vaccinated. Due to demographic changes (births, ageing, deaths, and immigrations) and due to individual changes in the risk status, individuals may change their age group or risk status during the vaccination period. If this happens, the memorized vaccination statistics is changed accordingly, so that the exact numbers of yet vaccinated and of yet unvaccinated individuals are known at any time during the vaccination period.

For an approximate calculation of the OR of revaccination, we neglect deaths and immigrations and assume that the vaccination coverage in the two subsequent years is the same (i.e. *N*^*+*^_*a,r*_ *= c*_a,r *_N_*a,r*_, and *N*^*−*^_*a,r*_ *= (1-c*_*a,r*_)_*_N_*a,r*_*)*, the relationship between the revaccination factor and the odds ratio (OR) of being vaccinated again can be approximately calculated as follows:

$$\begin{array}{ll} \mathrm{OR} & =\frac{\left(c_{a,r}f_{\mathrm{rev}}c_{a,r}\right)\;z\;\left(1-c_{a,r}+\left(f_{\mathrm{rev}}-1\right)c_{a,r}\right)\;z}{\left(c_{a,r}f_{\mathrm{rev}}\left(1-c_{a,r}\right)\right)\;z\;\left(c_{a,r}f_{\mathrm{rev}}\left(1-c_{a,r}\right)\right)\;z}\\ & =\frac{f_{\mathrm{rev}}\left(1-c_{a,r}+\left(f_{\mathrm{rev}}-1\right)c_{a,r}\right)}{1-c_{a,r}}, \end{array}$$

whereby *z = N*^*2*^_*a,r*_*/(N*^*−*^_*a,r*_ *+ f*_rev*_N^*+*^_*a,r*_*)*. Using the standard parameter value of *f*_*rev*_ *= 2*, we get OR = *2/(1-c*_*a,r*_*)*. For a vaccination coverage of 20%, the OR is 2.5, for 60%, it is 5.0.

## Appendix 6 Drift variants, vaccine mismatch, and cross protection

Smith *et al.*[35] constructed an antigenic map of Influenza A(H3N3) data from 35 years of surveillance (1968 to 2003) and found 11 antigenically distant clusters; the average antigenic distance between the centers of consecutive clusters was 4.5 units (SD 1.3). As influenza vaccines are updated when there is an antigenic difference of at least 2.0, these clusters can be regarded as representing genetically relevant drift variants. As ten *new* drift variants occurred in the time period of 35 years, we get an average duration of *D* = 3.5 years for new A(H3N2) drift variants (95% CI from 2.2 to 6.8). No direct estimates for the duration between consecutive Influenza B drift variants are available, but as they change at a rate which is about two to three times lower than this [3], we assume the average sojourn time of Influenza B drift variants to be 7 years. Judging by the frequency of changes in vaccine composition from 1987 to 2000, the variability of A(H1N1) may be similar to that of Influenza B [3]; we, therefore, use the same average duration of 7 years for Influenza A(H1N1) drift variants. For each one of the two A subtypes and the two B lineages, an independent random number *r* is calculated at the beginning of each simulation year to determine whether the circulating variant is replaced by a new drift variant. If *r < 1/D*, a new drift variant is introduced.

The possibility of a vaccine design mismatch is only considered for years in which new drift variants appear. Of course, TIV vaccination can only be affected by an Influenza B mismatch, if the mismatched B lineage is actually contained in the vaccine. For the last 18 years, four mismatch events have been reported (A(H3N2) in 2003 and 2004, and A(H1N1) in 2006 and 2009 [42]). For this time period, we calculate that 18/3.5 = 5.14 new drift variants for A(H3N2) and 18/7 = 2.57 new drift variants for A(H1N1) appeared. For the B lineage which was included in TIV during the 18 years, we also calculate 18/7 = 2.57 new drift variants. As we expect altogether 10.28 drift events and observed 4 mismatch events, we calculate a mismatch probability of 40% per drift. We use this probability for all four influenza variants, including the two B lineages for which no vaccine design mismatch has been reported.

The immunity shared between a previously circulating variant and a new variant is not well known. We infer information on this shared immunity from vaccination studies summarized by Jefferson *et al.*[39]: well-matched TIV vaccination protects on average 73% of healthy adults, whereas mismatched TIV vaccination protects only 44%. We take the ratio of 44/73 = 60% also as indicator for the shared immunity between subsequent drift variants. 60% of the individuals who were immune against the previously circulating variant are also immune against the new drift variant, whereas the remaining 40% are susceptible to the new drift variant. For the sake of simplicity, we replace variants by new drift variants at the beginning of a simulation year (i.e. on July 1st). We do not allow co-circulation of the old and the new variant in the simulation, and we assume that a previously circulating variant will never be introduced again.

We furthermore use this estimate of 60% for shared immunity between the two B lineages: if an individual is infected with one B lineage, an additional immunization (or booster event) with respect to the other lineage takes place with a probability of 60%. Likewise, TIV vaccination can cause immunity (or booster events) against the B lineage which is not contained in the vaccine; the age-dependent vaccine efficacy is multiplied by 0.6 to obtain the efficacy against the missing lineage. We restrict this cross-immunizing vaccination effect to TIV because there is no convincing evidence that increasing the antigen amount enhances the efficacy of influenza vaccines [57-59]. Tricco *et. al.*[10] report for vaccinees from 18 to 49 years of age that the vaccine efficacy against Influenza B is 77% if the lineage is contained in TIV and 52% if it is not contained in TIV, respectively. The ratio 52/77 = 0.675 indicates a B lineage cross protection of 67.5%. Langley *et al*. examine in a vaccination study with children what percentages seroconvert with respect to the B lineage which is contained and which is not contained in TIV. As they use two different TIV vaccines which either contain B/Vic or B/Yam, they obtain four different sero-conversion rates with respect to the Influenza B lineages [59] (summarized in Table 3). We have developed a maximum likelihood model based on the assumption that either one of the two TIV vaccines causes a direct sero-conversion *S* with respect to the B lineage contained in the vaccine and an indirect effect *S x* with respect to the lineage not contained in the vaccine. The joint likelihood *L* of the observations is then given by $L=\left(\begin{array}{c} 870\\ 359 \end{array}\right){\left(\mathrm{Sx}\right)}^{359}{\left(1-\mathrm{Sx}\right)}^{511}\left(\begin{array}{l} 870\\ 622 \end{array}\right)S^{622}{\left(1-S\right)}^{248}\left(\begin{array}{l} 877\\ 644 \end{array}\right)S^{644}{\left(1-S\right)}^{233}\left(\begin{array}{l} 877\\ 262 \end{array}\right){\left(\mathrm{Sx}\right)}^{262}{\left(1-\mathrm{Sx}\right)}^{615}$, omitting the constant factors, this simplifies to *L* ∝ (*Sx*)^359 + 262^(1 − *Sx*)^511 + 615^*S*^622 + 644^(1 − *S*)^248 + 233^.

The maximum likelihood estimates are *S* = 72.5% (95% confidence interval CI: 70.3-74.5, based on the profile likelihood) and *x* = 49.1% (CI: 45.7-52.5), respectively, i.e. the sero-conversion rate of the B lineage not contained in TIV is about half as high as that of the lineage which is contained in the vaccine. Having obtained three indicators for the amount of B lineage cross protection (49.1% Langley, 60% Jefferson and 67.5% Tricco, respectively), we have decided to use the value 0.6 as the baseline value in our simulation studies.

**Table 3:** **Sero-conversion rates as reported by Langley et al. **[59]**, defined as the proportion of vaccinees with a pre-vaccination titer <1:10 and a post-vaccination titer ≥1:40, or a pre-vaccination titer ≥1:10 and at least a 4-fold increase in post-vaccination titer**

**TIV vaccination group**	**Seroconversion rate**	
	**With respect to B Yamagata**	**With respect to B Victoria**
TIV-Vic (n = 870)	41.3% (n = 359)	71.5% (n = 622)
TIV-Yam (n = 877)	73.4% (n = 644)	29.9% (n = 262)

Abbreviations: TIV-Vic, trivalent influenza vaccine with Victoria lineage B strain; TIV-Yam, trivalent influenza vaccine with Yamagata lineage B strain. The numbers of sero-converters (n) were calculated from the percentages.

## Appendix 7 Results of TIV and QIV vaccination

**Figure 9:**
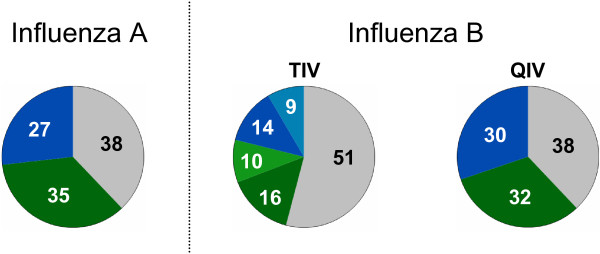
**Immunologic results of about 500,000 TIV and QIV vaccinations which are performed in the 20 year evaluation period of a randomly selected simulation.** The pie charts display the percentages of vaccinees who are immunized (green), boostered (blue) or fail to respond immunologically (grey). Immunologic responses with respect to A(H1N1) and A(H3N2) are averaged as “Influenza A”, and immunologic responses with respect to B/Vic and B/Yam are averaged as “Influenza B”. As TIV only contains one B lineage, the immunologic response to the missing lineage is either missing (grey) or it is caused by a cross immunization (light green) or cross-immunizing booster event (light blue).

During the 20 years of each simulation’s evaluation period, about half a million TIV or QIV vaccinations are performed in a population of 80,000 individuals. Due to the efficacy of the vaccine, about 38 percent of all vaccinations remain without immunologic effect (Figure 9). As many individuals are already immune when they are vaccinated, nearly a third of the successful vaccinations can only booster existing immunity. On average, 35% of all vaccinees newly acquire immunity against Influenza A. As TIV only contains one B lineage, the effect on Influenza B would only be half of that if no cross-immunizing events occurred. Due to cross-immunizing vaccination effects (light green and light blue areas), the effect of TIV vaccination is less pessimistic (Figure 9, middle). Using QIV, the failure rate drops to 38% (conveyed by an overall vaccine efficacy of 62%).

## Appendix 8 Sensitivity analyses

In the following, we present the results of sensitivity analyses: the number of infections with and without vaccination is first accumulated for the 20 year evaluation period of each simulation and divided by 20 to obtain mean numbers of infections per simulation year. These means are used to calculate overall averages and 95% confidence intervals (given in Tables 4, 5, 6, 7 and 8). Pair-wise differences between the TIV scenario and the QIV scenario of each simulation allow calculating the number of influenza infections prevented by TIV. The values in the tables are arithmetic means (averaged over 1,000 or 2,000 simulations) and their 95% confidence intervals. Please note that the percentages reported in Tables 4, 5, 6, 7 and 8 are calculated as arithmetic means of the percentages observed in the individual simulations. Calculating the percentages from the mean numbers of infections yield slightly different results: for the baseline parameter setting (R0 = 1.575 in Table 4), the calculation based on the mean values would yield 100%*395/(5597 + 3346) = 4.4% of infections prevented by QIV whereas the average percentage (calculated from 2,000 simulations) yields 4.3%.

**Table 4:** **Sensitivity analysis exploring the influence of the basic reproduction number R**_**0**_

***R***_***0***_	**Influenza A**		**Influenza B**			**Prevented**	
	**No vaccination**	**TIV or QIV**	**No vaccination**	**TIV**	**QIV**	**QIV-TIV**	**%**
1.2	5880	1954	3054	1400	1075	325	9.5
	(5823–5936)	(1923–1985)	(3018–3089)	(1379–1422)	(1056–1095)	(306–344)	(8.9-10.1)
1.3	7221	3061	3754	1999	1626	373	7.3
	(7151–7290)	(3018–3104)	(3709–3798)	(1970–2028)	(1599–1652)	(350–396)	(6.8-7.7)
1.4	8359	4106	4363	2541	2167	374	5.5
	(8276–8442)	(4055–4158)	(4311–4415)	(2506–2577)	(2133–2201)	(350–398)	(5.2-5.9)
1.5	9302	5009	4889	3063	2677	386	4.8
	(9209–9395)	(4946–5071)	(4830–4947)	(3021–3105)	(2638–2717)	(360–411)	(4.4-5.1)
**1.575**	**9820**	**5597**	**5229**	**3346**	**2951**	**395**	**4.3**
	**(9750–9891)**	**(5550–5643)**	**(5183–5275)**	**(3313–3379)**	**(2920–2982)**	**(376–414)**	**(4.1-4.5)**
1.6	10154	5787	5345	3482	3086	396	4.2
	(10051–10256)	(5716–5858)	(5280–5410)	(3435–3529)	(3041–3132)	(370–422)	(3.9-4.5)
1.7	10893	6510	5736	3880	3469	411	3.9
	(10784–11002)	(6433–6587)	(5664–5809)	(3826–3934)	(3419–3518)	(385–437)	(3.7-4.2)
1.8	11494	7116	6060	4218	3820	398	3.5
	(11374–11614)	(7032–7200)	(5982–6138)	(4160–4277)	(3765–3875)	(372–424)	(3.3-3.7)
1.9	12063	7669	6383	4523	4139	383	3.1
	(11935–12190)	(7578–7760)	(6301–6466)	(4461–4584)	(4081–4198)	(357–409)	(2.9-3.3)
2.0	12354	8183	6640	4790	4404	385	3.0
	(12227–12480)	(8087–8279)	(6550–6731)	(4724–4855)	(4342–4467)	(359–411)	(2.8-3.2)

Average annual results (with 95% confidence intervals) obtained for the 20 year evaluation period (calculated from 1,000 simulations for each combination of *R*_*0*_ and vaccination strategy in a simulated population of about 80,000 individuals (2,000 simulations each for the baseline value of *R*_*0*_ = 1.575; boldface line). The last column shows what percentage of all influenza A and B infections which occur under TIV vaccination can additionally be prevented by QIV.

**Table 5:** **Sensitivity analysis exploring the influence of the duration of naturally acquired immunity D**_**I**_

***D***_***I***_	**Influenza A**		**Influenza B**			**Prevented**	
	**No vaccination**	**TIV or QIV**	**No vaccination**	**TIV**	**QIV**	**QIV-TIV**	**%**
4	12644	6965	6743	4302	3785	517	4.6
	(12548–12740)	(6896–7035)	(6681–6805)	(4256–4349)	(3741–3829)	(490–545)	(4.3-4.8)
**6**	**9820**	**5597**	**5229**	**3346**	**2951**	**395**	**4.3**
	**(9750–9891)**	**(5550–5643)**	**(5183–5275)**	**(3313–3379)**	**(2920–2982)**	**(376–414)**	**(4.1-4.5)**
8	8482	4944	4474	2924	2595	329	4.1
	(8385–8579)	(4878–5010)	(4410–4539)	(2879–2970)	(2552–2638)	(305–354)	(3.8-4.4)
10	7682	4524	4055	2631	2363	267	3.7
	(7585–7780)	(4460–4589)	(3990–4119)	(2585–2676)	(2320–2406)	(243–291)	(3.3-4.0)
12	7209	4259	3766	2476	2194	282	4.1
	(7110–7307)	(4195–4323)	(3702–3831)	(2432–2520)	(2152–2236)	(257–306)	(3.7-4.5)

Average annual results (with 95% confidence intervals) obtained for the 20 year evaluation period (calculated from 1,000 simulations for each combination of *D*_*I*_ and vaccination strategy in a simulated population of about 80,000 individuals (2,000 simulations each for the baseline value of 6 years; boldface line). The immunity loss rate (baseline value 1/(9.13 years)) is varied to obtain the average duration of immunity given in the *D*_*I*_ column; these values apply to A(H1N1) and to the B lineages. Due to the higher drift frequency, the resulting durations for A(H3N2) are lower: 3.2 years, 4.5 years, 5.5 years, 6.4 years and 7.1 years, respectively. The last column shows what percentage of all influenza A and B infections which occur under TIV vaccination can additionally be prevented by QIV.

**Table 6:** Sensitivity analysis exploring the influence of the degree of Influenza B lineage cross protection L

***L***	**Influenza B**			**Prevented infections**	
	**No vaccination**	**TIV**	**QIV**	**QIV-TIV**	**%**
0%	8283	6552	4372	2180	17,9
	(8224–8394)	(6477–6627)	(4313–4431)	(2142–2217)	(17,6-18,2)
10%	7557	5760	4009	1751	15,4
	(7491–7649)	(5692–5827)	(3954–4063)	(1716–1786)	(15,1-15,7)
20%	6961	5140	3772	1368	12,7
	(6890–7037)	(5079–5200)	(3720–3824)	(1336–1399)	(12,5-13,0)
30%	6491	4624	3574	1050	10,2
	(6445–6587)	(4569–4680)	(3526–3623)	(1020–1080)	(10,0-10,5)
40%	6144	4216	3386	830	8,4
	(6070–6206)	(4163–4269)	(3339–3433)	(801–859)	(8,1-8,7)
50%	5837	3865	3276	589	6,2
	(5783–5916)	(3815–3914)	(3230–3322)	(562–615)	(5,9-6,5)
**60%**	**5229**	**3346**	**2951**	**395**	**4.3**
	**(5183–5275)**	**(3313–3379)**	**(2920–2982)**	**(376–414)**	**(4.1-4.5)**

Average annual results (with 95% confidence intervals) obtained for the 20 year evaluation period (calculated from 1,000 simulations for each combination of *L* and vaccination strategy in a simulated population of about 80,000 individuals (2,000 simulations each for the baseline parameter value *L* = 60%; boldface line). The last column shows what percentage of all influenza A and B infections which occur under TIV vaccination can additionally be prevented by QIV.

**Table 7:** Sensitivity analysis exploring the influence of the duration of indirect protection against infection

**z**	**Annual mean number of infections with TIV**	**Annual mean number of infections with QIV**	**Annual mean number of infections prevented by QIV**	**Percentage prevented**
**0**	**8943**	**8548**	**395**	**4.3**
	**(8885–9001)**	**(8491–8604)**	**(376–414)**	**(4.1-4.5)**
7	9024	8617	407	4.3
	(8948–9100)	(8541–8694)	(367–447)	(3.9-4.8)
14	9176	9727	449	4.7
	(9097–9254)	(8652–8802)	(409–488)	(4.2-5.1)
21	9186	8722	465	4.9
	(9114–9259)	(8649–8794)	(424–505)	(4.5-5.3)
28	9151	8732	420	4.3
	(9077–9226)	(8660–8804)	(378–461)	(3.9-4.8)

Average annual results (with 95% confidence intervals) obtained for the 20 year evaluation period (calculated from 1,000 simulations for each combination of the unspecific protection and vaccination strategy in a simulated population of about 80,000 individuals (2,000 simulations each for the baseline value indicated by z = 0; boldface line). Indirect protection begins 1 day after infection and ends z days after infection; it prevents infection with any one of the influenza viruses. The last column shows what percentage of all influenza infections which occur under TIV vaccination can additionally be prevented by QIV.

**Table 8:** Sensitivity analysis exploring the influence the contact network turnover

**Network turnover**	**Annual mean number of infections with TIV**	**Annual mean number of infections with QIV**	**Annual mean number of infections prevented**	**Percentage prevented by QIV**	**Contacts persist for 40 years**
Lower	9039	8596	442	4.9	0.030%
	(8955–9123)	(8513–8680)	(416–470)	(4.6-5.2)	
**Baseline**	**8943**	**8548**	**395**	**4.3**	**0.361%**
	**(8885–9001)**	**(8491–8604)**	**(376–414)**	**(4.1-4.5)**	
Higher	9135	8719	416	4.5	9.310%
	(9053–9218)	(8638–8800)	(390–442)	(4.2-4.8)	

Average annual results (with 95% confidence intervals) obtained for the 20 year evaluation period (calculated from 1,000 simulations for each combination of network turnover and vaccination strategy in a simulated population of about 80,000 individuals (2,000 simulations each for baseline turnover; boldface line). Higher network turnover is obtained by replacing a random sample of 10% of all contacts annually. Lower turnover is obtained by calling the “repair routine” which matches the simulated contact network with the POLYMOD matrix once a year instead of daily (this leads to larger and longer lasting differences between the simulated network and the POLYMOD matrix). The last column shows what percentage of the “childhood acquaintances” of 10 year old children at the beginning of the simulation are still among their contacts at the end of the simulation when they are 50 year adults.

## Abbreviations

4Flu: Name of the individual-based seasonal influenza simulation tool; TIV: Trivalent influenza vaccine; QIV: Quadrivalent influenza vaccine; CI: Confidence interval; OR: Odds ration; R0: Basic reproduction number; RR: Relative risk, WHO, World health organization; A(H1N1): Influenza A subtype H1N1; A(H3N2): Influenza A subtype H3N2; B/Yam: Influenza B, Yamagata lineage; B/Vic: Influenza B, Victoria lineage.

## Competing interests

ME is shareholder of Epimos GmbH & Co. KG, which has received research support from the GlaxoSmithKline group of companies and AstraZeneca. MS is shareholder of ExploSYS GmbH, which has received payments from Epimos GmbH & Co. KG for developing the simulation tool 4Flu. JH and RSO are employees of the GlaxoSmithKline group of companies, and have restricted shares, stocks and/or stock options ownership in the GlaxoSmithKline group of companies. BS has received consultancy fees and honoraria for presentations from the GlaxoSmithKline group of companies and Novartis. MK has received consultancy fees and honoraria for advisory activities and presentations from Novartis, the GlaxoSmithKline group of companies and AstraZeneca.

## Authors’ contributions

ME has created the transmission model and has written the major part of the manuscript. MS has designed and programmed the simulation tool and has taken part in the model development. JH and RSO participated to the structural design of the model and drafted parts of the introduction and the discussion section of the paper. HU, BS and MK have provided expert input on the epidemiology of seasonal influenza. They also have aided in the parameterisation and discussion of the model and have written parts of the manuscript. All authors had full access to the data and gave final approval before submission.

## Pre-publication history

The pre-publication history for this paper can be accessed here:

http://www.biomedcentral.com/1471-2334/14/365/prepub
